# Unraveling Excited State Dynamics of a Single-Stranded DNA-Assembled Conjugated Polyelectrolyte

**DOI:** 10.1021/acs.jpclett.3c01803

**Published:** 2023-10-26

**Authors:** Eliana Nicolaidou, Anthony W. Parker, Igor V. Sazanovich, Michael Towrie, Sophia C. Hayes

**Affiliations:** † Department of Chemistry, University of Cyprus, P.O. Box 20537, 1678 Nicosia, Cyprus; ‡ Central Laser Facility, Research Complex at Harwell, Science and Technology Facilities Council, Rutherford Appleton Laboratory, Harwell Oxford, Didcot, Oxfordshire OX11 0QX, U.K.

## Abstract

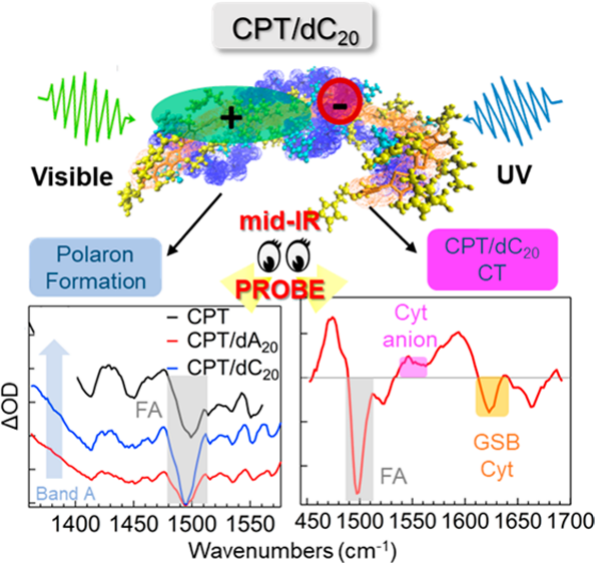

Conformational templating of conjugated polyelectrolytes with single-stranded DNAs (ssDNAs) has the prospect of tailoring excited state dynamics for specific optoelectronic applications. We use ultrafast time-resolved infrared spectroscopy to study the photophysics of a cationic polythiophene assembled with different ssDNAs, inducing distinct conformations (flexible disordered structures vs more rigid complexes with increased backbone planarity). Intrachain polarons are always produced upon selective excitation of the polymer, the extent being dependent on backbone torsional order. Polaron formation and decay were monitored through evolution of IR-active vibrational modes that interfere with mid-IR polaron electronic absorption giving rise to Fano-antiresonances. Selective UV excitation of ssDNAs revealed that stacking interactions between thiophene rings and nucleic acid bases can promote the formation of an intermolecular charge transfer complex. The findings inform designers of functional conjugated polymers by identifying that involvement of the scaffold in the photophysics needs to be considered when developing such structures for optoelectronic applications.

DNA can be seen as a scaffold for tuning the optoelectronic properties of conjugated polymers when appropriate interactions during self-assembly direct their backbone conformation. This DNA-based approach relies on the strong correlation of photophysical properties with polymer backbone conformation.^1−4^ Understanding the nature of the structure of the complex and the way it influences the photophysical properties makes studying both their ground and excited state properties by structurally probing each partner of equal importance. A deeper fundamental understanding of ultrafast photoinduced processes is of utmost importance to further advance the molecular design of such nanostructures, since optoelectronic functionality is governed deeply by the formation and decay dynamics of charged and neutral excitations.

Among π-conjugated polymers, cationic π-conjugated polyelectrolytes facilitate the complexation with nucleic acids through electrostatic interactions of positively charged polymer side chains with the negatively charged phosphodiester groups in the nucleic acid backbone.^5^ An intriguing example is the cationic polythiophene polymer (CPT) with imidazolium side chains (Scheme 1).^6−8^ According to our previous work,^9^ this polymer can adopt an extended polythiophene chain when assembled with homonucleotide oligocytosine (dC_n_) strands, following a mechanism of complexation that involves extensive π-stacking interactions between thiophene and cytosine rings, further enhanced by π-stacking with the imidazolium cation, that leads to improved backbone planarization. On the contrary, complexation with oligoadenosine (dA_20_) through primarily electrostatic interactions leaves the polymer backbone more flexible and torsionally disordered.

**Scheme 1:**
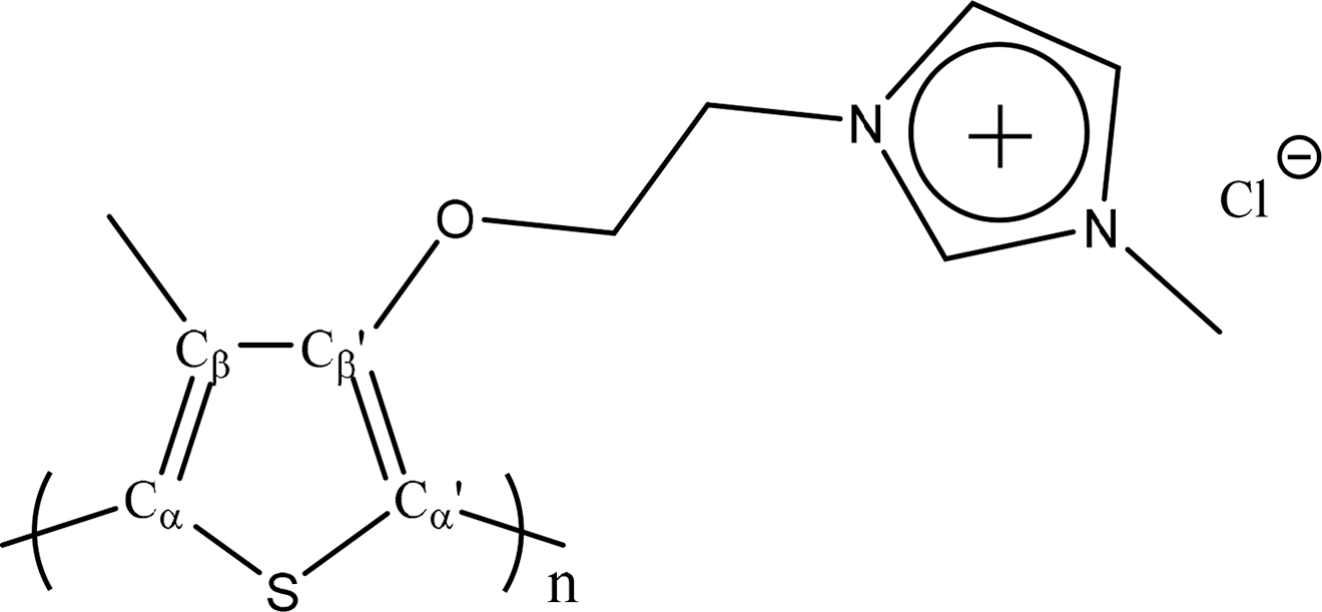
Molecular Structure of Cationic Poly(1*H*-imidazolium, 1-methyl-3-[2-[(4-methyl-3-thienyl)oxy]ethyl]-, chloride) (CPT)

Optically excited charge carriers on a π-conjugated chain display absorption bands in the near-IR and mid-IR due to new energy levels inside the optical band gap compared to their ground state counterparts.^10^ These polaron bands have been detected experimentally for a range of polymers through a quasi-steady state measurement, known as photoinduced absorption (PIA).^11^ Spectroscopically in the near-IR region, polaron band P_2_, corresponding to the transition from the HOMO to the highest polaron energy level in the gap,^12−15^ is detected in the case of polythiophenes at ∼10000 cm^–1^ (1000 nm) and formation after optical excitation can therefore be monitored through transient absorption (TA) spectroscopy. The intensity of P_2_ is a key indicator of the existence of interactions between different polymer chains (interchain interactions), exhibiting moderate intensity when interchain interactions are weak.^12,13^ TA measurements on CPT alone^16^ and on CPT/ssDNA^9^ in solution were conducted in our previous studies. CPT alone favors the generation of long-lived polarons at low temperatures and triplets at higher temperatures, detected in the near-IR spectral region.^16^ However, when CPT is complexed with ssDNAs,^9^ regardless of the sequence, the photophysical behavior is comparable, described by the formation of excitons with similar dynamics. This raises the question as to why the complexation that induces different polymer conformations does not seem to affect the excited state behavior of the polymer, or whether the TA measurements simply could not detect some other excited species formed due to the spectral range investigated.

In the mid-IR range of PIA of positively charged polythiophene chains, two bands are observed known as A and B. Band A (also referred to as a combination of the delocalized polaron band (DP_1_) or charge transfer (CT)^17^ and P_0_ band^18,19^) is composed of overlapping interchain and intrachain components. One component can dominate over the other, depending on the effectiveness of polaron delocalization over several chains due to significant interchain coupling, or along the polymer (intrachain) in the presence of noninteracting chains.^20,21^ Band B (also known as P_1_) corresponds to the transition from the HOMO to the lowest polaron energy level in the gap.^13,15,20^ According to the PIA spectra, the polaron bands A and B can be detected at ∼1000 and 4000 cm^–1^, respectively.^10,21^ Therefore, band A can overlap with strong vibrational IR bands, which are originally Raman-active (A_g_) modes coupled to the π-electron system that become strongly absorbing infrared-active vibrations, referred to as IRAVs. IRAVs occur through structural distortion stemming from localized charge carriers on the π-conjugated chain creating large changes in bonding dipole moments.^22−25^ When overlap of IRAV modes and polaron electronic absorption exists, Fano antiresonances (FA) emerge.^11,21,26−29^ The FA bands can be interrogated by time-resolved IR (TRIR), enabling monitoring of the photogenerated charged species and simultaneously accessing crucial information on structural evolution that occurs during the rapid relaxation processes,^29,30^ often inaccessible by conventional spectroscopy. In particular, this was clearly demonstrated through picosecond time-resolved photoinduced IRAV studies of Mizrahi et al.^31,32^ and Miranda et al.,^33^ by selectively detecting photogenerated polarons using IRAV modes of PPV polymer chains as a direct measure, avoiding interference with overlapping bands from neutral excitations, such as excitons and excimers as is the case with transient absorption measurements in the visible/near/IR. Similar studies on polythiophene films, enabled monitoring of polaron diffusion, charge carrier separation, and recombination, as well as detection of structural modifications attributed to polaron relaxation processes of the polymer.^34,35^

Here, we employed TRIR in the mid-IR as a structural probe to compare the excited state behavior of a loosely bound CPT/DNA-base complex with a more rigid and ordered system. We report the results from a series of experiments comparing the excited state dynamics for solutions of the pure compounds and their corresponding complexes in deuterated PBS buffer through selective excitation of each compound at 532 and 266 nm, coinciding with the polymer and oligonucleotide absorption, respectively. Results from both excitation wavelengths were dependent on the strength and nature of interactions that are strongly controlled by the sequence of ssDNA. While intrachain polarons are formed in both complexes following excitation at 532 nm, close interaction of the polymer with the ssDNA perturbs the environment of the stacked base, which is clearly indicated with excitation of the ssDNA by the formation of a charge-transfer complex between the two components.

We begin by describing our results showing the TRIR spectra of CPT alone with excitation at 532 nm (Figure 1a). A prominent feature is the large contribution from background absorption (appearing as a baseline offset) to the spectra, clearly observed at the earliest delay times, decreasing steadily with time and seeming to correlate with the decrease in IR mode intensities. We assign this background to an electronic absorption due to polaron formation within the instrument response (∼200 fs), supported by the observation of polaron absorption in the near-IR TA spectra of CPT in the ordered phase reported previously,^16^ corresponding to the P_2_ polaron band (10470 cm^–1^, 955 nm). The background absorption observed in the TRIR spectra is therefore attributed to band A, which is expected to be located in the ∼800–1600 cm^–1^ region according to the rr-P3HT literature,^19,20^ coinciding with the spectral region we collected the spectra. We can conclude here that in the absence of ssDNA, excitation of ordered CPT chains at 532 nm (as seen in our previous work on CPT with resonance Raman spectroscopy,^9^ where excitation at this wavelength probes the more planar polymer chain segments) leads to intra- and interchain polaron delocalization.

**Figure 1:**
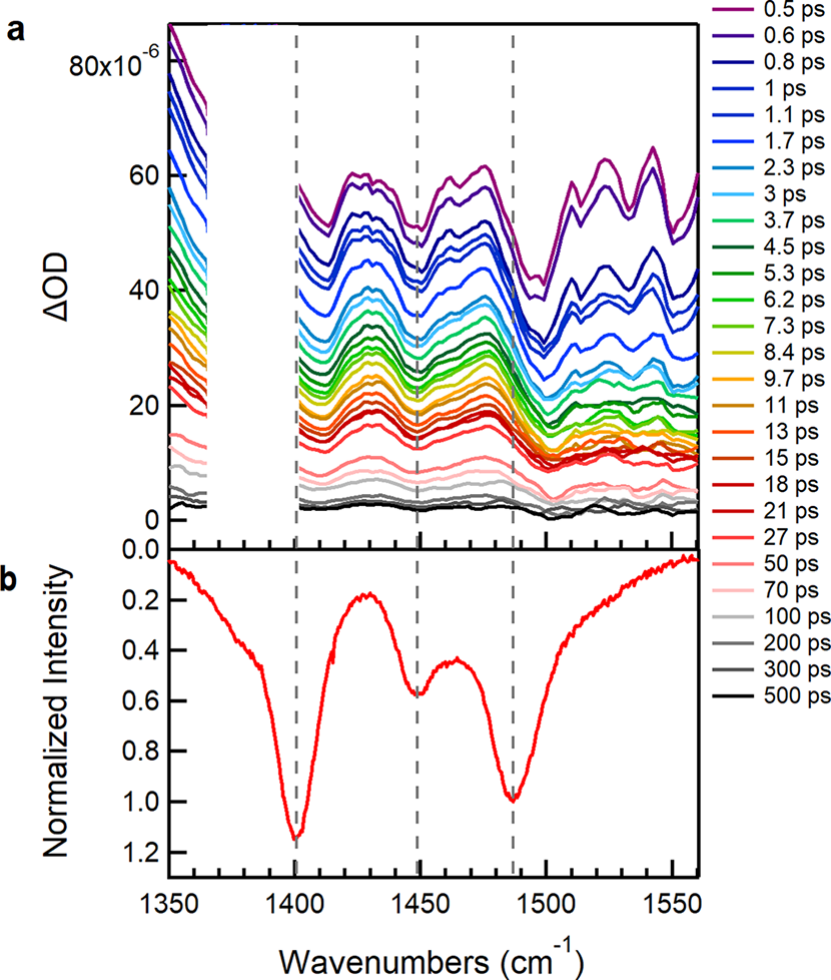
(a) TRIR spectra of CPT following excitation at 532 nm. Note: the spectral region 1370–1400 cm^–1^, is dominated by an experimentally induced artifact feature and has been omitted for clarity. No extra vibrational features are expected in this region as seen in Figures 2a and S1a). (b) Ground state Raman spectrum of CPT with excitation at 473 nm with inverted *y* axis.

Generally, the existence of such a broad background hinders the discrimination of vibrational ground state bleaches and transient bands (negative and positive bands, respectively). Nonetheless, we observe significant spectral dips in the TRIR spectra at wavenumbers that are similar to the resonance Raman spectra of the ground state (Figure 1b). The intensity of these bands is strongly indicative of IRAV modes, which here spectrally coincide with the electronic absorption band A, and therefore, these dips are attributed to Fano-antiresonances.^19,27,28,36^ This concept is also evidenced in the TRIR spectra of P3HT that we collected (Figure S1c), for which photoinduced absorption in the mid-IR spectral range is widely reported in the literature, facilitating the interpretation of the data. The similarity of the P3HT PIA spectrum accompanied by apparent Fano-antiresonances (Figure S 1d, reproduced from the literature) with the TRIR spectra provides strong validation for the occurrence of overlap between the electronic band and IRAV modes. The existence of coupling between the absorption background and the various vibrational signals is graphically demonstrated (Figure S2) by the linear relationship between the intensity of each vibrational dip and the intensity of a point in the spectra considered as the background at various delay times. The reduction in background absorption due to polaron recombination is correlated with the observed loss in intensity of the FA modes.

Similar behavior is observed in the TRIR spectra of the CPT/ssDNA complexes (Figure 2a and Figure S3) showing a broad background absorption signal, assigned to band A, and vibrational dips assigned to FA. In the case of CPT/ssDNA complexes, TA spectroscopy was unable to show polaron formation, since in the spectral region accessed the only indication of the existence of polarons is the P_2_ polaron band, which was not evident in any of the TA spectra of the complexes. Nonetheless, the absence of the P_2_ band is a key sign for weak interchain interactions and suggests that the intrachain contribution in band A of the TRIR spectra of the complexes is stronger.^18−21^ Therefore, we can deduce that when CPT is assembled with ssDNA, this is nonaggregated, confirming that the interactions between ssDNA with CPT are strong enough to isolate a single semiconducting polymer chain.

**Figure 2:**
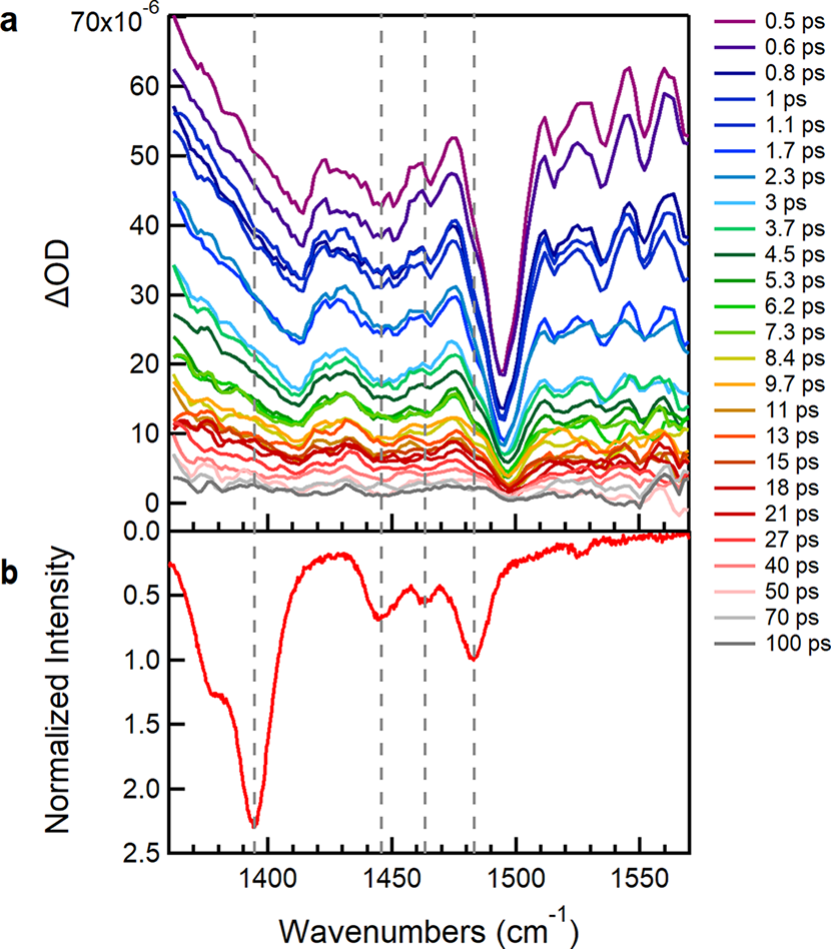
(a) TRIR spectra of CPT/dC_20_ with excitation at 532 nm. (b) Ground state Raman spectrum of CPT/dC_20_ with excitation at 532 nm with reversed *y* axis.

Figure 3 compares TRIR spectra of CPT and those when complexed with two different ssDNAs, dA_20_, and dC_20_. We observe a significant difference in the intensity of the background absorption between the two ssDNAs (Figure 3). The ∼30% decrease in the background absorption signal in CPT/dA_20_ can be attributed to the increased disorder of the backbone conformation that leads to attenuation of the oscillator strength for band A,^14^ as well as to the fewer ordered (more planar) polymer chain segments excited at 532 nm, according to the absorption spectrum of CPT in this complex (Figure S4a).^9^ These more torsionally ordered backbone segments are expected to contribute the most to the TRIR spectra shown herein. In contrast to the signal in CPT/dA_20,_ the increased electronic absorption of CPT/dC_20_ is attributed to intrachain order (i.e., greater planarity), while in the case of CPT alone to interchain order.^14^ The fact that CPT alone is characterized by increased torsional order compared to CPT/dA_20_ is demonstrated by the comparison of the respective ground state resonance Raman spectra shown in Figure S4b, where a greater ratio between the C–C and C=C symmetric stretching modes of the thiophene ring is observed in the case of CPT alone.^9^

**Figure 3:**
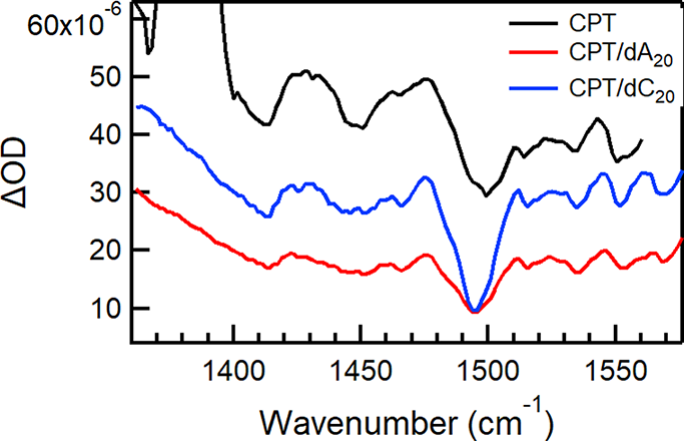
TRIR spectra at 1 ps delay of CPT (black), CPT/dA_20_ (red), and CPT/dC_20_ (blue). The latter spectrum was divided by 1.25 to adjust for the concentration differences.

The intensity of IRAV modes is linked to the extent of polaron delocalization along the polymer chain and the associated charge displacement during vibrational motion, as well as the proximity with a low-lying electronic transition leading to strong vibronic coupling.^29,37^ Increased delocalization of positive charge carriers is evidenced by higher intensity.^23,24^ Therefore, the largest intensity of the FAs in CPT/dC_20_ (Figure 3) is associated with a more delocalized intrachain polaron. The dramatic change in the 1498 cm^–1^ FA intensity, assigned to the C=C symmetric stretching of the thiophene ring,^9^ is attributed to the sensitivity of this mode to changes in charge density during the oscillation that displace the polaron along the long axis of the conjugated molecule generating an increased dipole moment with large variations.^23,36,38^ This means, that in the case of CPT/dC_20_, both the largest population of polarons and the greatest delocalization length is derived among the samples tested, which is facilitated by the increased degree of planarity of the polymer backbone.^9^ As seen above, the intensity of the FA bands also varies with the intensity of the broad absorption background, which could contribute to the reduced FA intensity in the CPT/dA_20_ complex. However, in the case of CPT alone with known reduced torsional order compared to CPT/dC_20_ (Figure S4b),^9^ the electronic absorption background is significant due to contributions to the electronic band from interchain interactions, while the 1498 cm^–1^ FA lacks intensity, representative of a reduced intrachain exciton delocalization compared to CPT/dC_20_.

In addition to the intensity, the position of IRAVs can be sensitive to the π-electron configuration due to the strong electron–phonon coupling.^23^ The position of IRAVs reported in the literature usually stems from quasi-steady state spectra obtained either through PIA or charge modulation spectroscopy (CMS) or from FTIR spectra of chemically/electrochemically doped samples. TRIR, as a direct probe for polarons, reveals the time scale for their formation and decay through monitoring the evolution of the IRAV modes post photoexcitation and provides insights on the excited state processes at play. Interestingly, in all cases, TRIR spectra at early times (e.g., 300 fs) display a prominent shift between the C_β_–C_β_ and C_α_=C_β_(Me) (1414 and 1498 cm^–1^_,_ respectively) symmetric stretching IRAV bands (Figure 1a, 2a and S3a) and the corresponding resonance Raman (RR) bands (1400 and 1487 cm^–1^ respectively, Figure 1b, 2b and S3b) with excitation either at 473 or 532 nm.^9^ The position of the C_β_-C_β_ stretch band is harder to determine accurately due to its broadness and asymmetric shape. However, focusing on the C_α_=C_β_ stretch, the position at 1498 cm^–1^ is reminiscent of the position in the resonance Raman spectrum recorded for the neutral CPT with 405 nm excitation,^16^ i.e., for CPT chains that are torsionally disordered, with reduced conjugation length. As previously noted in the literature,^23,27^ the density of photoinduced charge carriers is smaller than what can be chemically produced. Therefore, the polymers can host both neutral and charged segments. The structural distortion due to polaron formation reduces the conjugation length of these planar charged segments, causing a blue shift (higher wavenumber) in the position of the IR-activated C_α_=C_β_ stretch.

At later delay times the 1498 cm^–1^ FA band upshifts, but to a different extent and with different dynamics in CPT alone relative to the CPT/dA_20_ and CPT/dC_20_ complexes_._ This C=C symmetric stretching band is known to be sensitive to chain conformation.^9,27,39−41^ Polaron localization accompanied by conformational relaxation could be at the origin of the shift.^42^ The smallest shift is observed in the case of CPT/dC_20_ (3 cm^–1^), compared to that of CPT/dA_20_ (7 cm^–1^) or CPT alone (9 cm^–1^). This reflects the rigidity of the CPT/dC_20_ complex, allowing for the smallest conformational reorganization (bond length change). This observation follows the same trend with the total exciton reorganization energy (λ_*tot*_) calculated for the complexes in our previous work using Resonance Raman Intensity Analysis (RRIA) (λ = 1119 cm^–1^ (0.139 eV) for dC_20_, and 3024 cm^–1^ (0.375 eV) for dA_20_). In that work, simultaneous fitting of the absorption and absolute resonance Raman cross sections provided access to the displacements, Δ, between the ground and excited state potential energy surface minima in each vibrational mode ω_*i*_, and thus to the mode-specific reorganization energies, λ_*i*_.^9^ Thus, λ_*tot*_ reflects the overall magnitude of the conformational rearrangement in the excited state and subsequently the rigidity of the chain conformation. This argues that conformational relaxation is influenced by the rigidity that characterizes each case. Such a trend in reorganization energy was previously observed for a series of donor–acceptor polymers with different amounts of energetic disorder; larger exciton as well as charge transfer reorganization energies were observed for the polymers with the largest disorder.^43^ This trend is also reflected here in the time scale of the observed shift (Figure S5). In CPT alone, the full extent of the shift occurs in the first 8 ps (time constant 1.2 ± 0.4 ps), after which no more changes are observed except reduction in the intensity of the band due to recombination. In CPT/dA_20_ where electrostatic interactions with the ssDNA limit relaxation to some extent, spectral evolution of this band occurs over 30 ps (time constant 19 ± 4 ps), albeit to a lesser extent than CPT alone, while in CPT/dC_20_, with extensive interactions with the ssDNA bases, minimal evolution (2–3 cm^–1^) occurs over the first 40 ps.

Moreover, fitting of the dynamics of the ∼1498 cm^–1^ band intensity (after elimination of the background contribution, see Figure S6) with a biexponential model gives average time constants for CPT alone of 24 ± 6, similar to the case of the CPT/dC_20_ complex (22 ± 7 ps), whereas the same band for the CPT/dA_20_ complex decays significantly faster (15 ± 3 ps). While a longer-lived polaronic species (due to interchain interactions) was observed in the TA spectra at 950 nm in our previous study of CPT alone,^16^ which should follow the same dynamics as the species observed in the mid-IR, the shorter temporal observation window in the present experiments (0.5 ns) hinders direct comparison, even though a small offset is also observed here. However, the large majority of the signal (95%) decays by 200 ps, which we interpret as due to intrachain recombination, as this would explain the similarity of the recombination dynamics to the case of the CT/dC_20_ complex where interchain interactions are excluded. The faster recombination dynamics observed in the dA_20_ complex can be attributed to the polymer backbone disorder due to the limited interactions between CPT and the ssDNA, which limits carrier diffusion. Therefore, the similarity of the vibrational bands in the TRIR spectra of CPT alone with those of the complexes indicate their common origin, while the difference in lifetimes reflect the modification of the interactions (simultaneous intra- and interchain) that the polymer experiences that consequently affect its conformation. The difference in the lifetimes and the generally faster decay dynamics in the dA_20_ complex is also evident in the background absorption (polaron band A) kinetics confirming that the electronic and vibrational features belong to the same species (Figure S7 and Table S1).

To gain more information about the excited state dynamics of the complexes, we turn to focus on the second component of the complexes, the oligonucleotides, by conducting time-resolved IR measurements with excitation at 266 nm. While at this wavelength we expect excitation of the ssDNAs, the CPT absorption spectrum (Figure S4a) indicates that the polymer absorbs also in this spectral region, possibly contributing to the TRIR spectra. In the case of CPT/dC_20_, a very prominent feature of the TRIR spectra is background absorption (Figure 4a). The background is also observed in the TRIR spectra of CPT alone with UV excitation (Figure S8) but not of dC_20_ alone (Figure S9), and therefore, the background cannot be attributed to changes in the cytosine bases upon excitation. Thus, the background absorption in the case of CPT alone can be attributed as above to intra- and interchain polaron formation after excitation to higher and more delocalized electronic states.^40,44^ Additionally, both CPT and dC_20_ have comparable electronic absorption intensities at 266 nm, leading to complicated vibrational spectra consisting of signals from both components. To disentangle the contribution of each component, bands were assigned to dC_20_ in agreement with the literature,^45−47^ with the remaining bands attributed to the polymer. Assignment of the ground state bleaches was performed by considering both literature FTIR spectra as well as ATR-FTIR spectra taken from drop-casted films of dC_20_, CPT, and the complex (Figure S10). Even though some contribution from the water bend band at ∼1650 cm^–1^ overlaps the carbonyl region, the contribution from the base vibrations can still be discerned.

**Figure 4:**
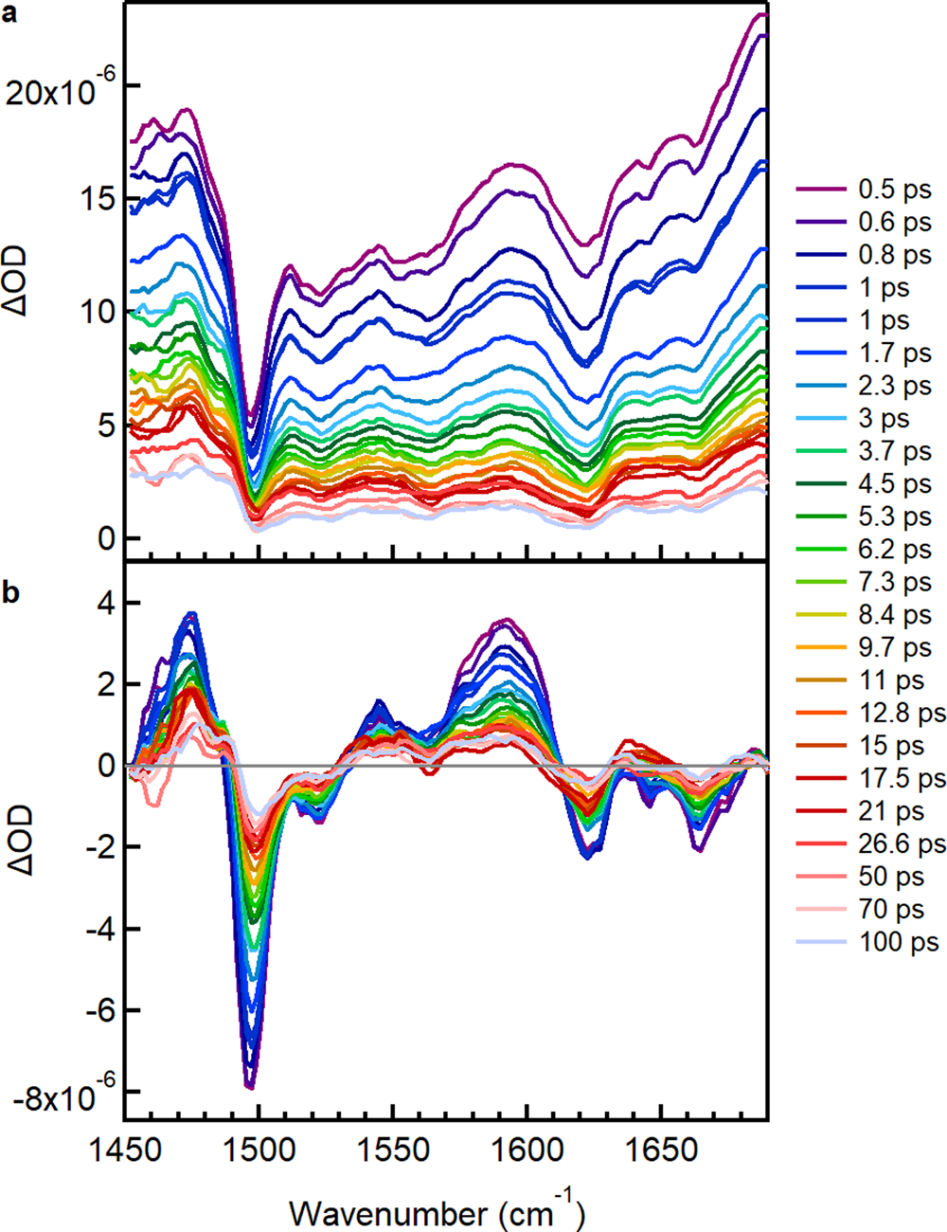
TRIR spectra of CPT/dC_20_ (a) before and (b) after background subtraction with excitation at 266 nm.

Subtracting the background absorption (Figure 4b) reveals rich vibrational signatures that evolve with time. Comparison of the TRIR spectrum of CPT/dC_20_ with the TRIR spectrum of dC_20_ alone at the same time delay (2 ps) (Figure S10) helps identify any bands that correspond to CPT, as well as detect the influence of the interaction of the two components on the ssDNA structure and dynamics. The TRIR spectra of the CPT/dC_20_ complex are significantly different from those of the oligonucleotide. Nonetheless, we can discern characteristic signatures of the base in the TRIR spectrum of CPT/dC_20_, both in the ground state bleaches and in the transient bands. It is important to note here that in the case of bands associated with dC_20_, the dips are considered as bleaches, i.e., as a decrease of ground state population, rather than Fano antiresonances as above, as confirmed by the similarity with the dC_20_ alone TRIR spectra and the ground state FTIR spectrum, and the fact that the electronic background absorption must be associated with the polymer, which thus remains uncoupled with the ssDNA vibrations. The ground state bleaches that relate to dC_20_ appear at 1520 cm^–1^, attributed to in-plane ring modes and at 1665 cm^–1^ due to the carbonyl group.^46^ As previously reported, the detection of carbonyl peaks at 1665 and 1695 cm^–1^, have been associated with polycytosine chains forming the i-motif structure.^46^ This requires careful consideration of our TRIR data; see Figure S10 where we observe spectral similarities of cytosine modes for the dC_20_ (pH 7) and literature spectra of dC_30_ in the i-motif structure at pH 5.5, observing modes at 1665 and 1700 cm^–1^.^46^ We do not expect dC_20_ to adopt the i-motif configuration as a dominant form at pH 7 and within the CPT/dC_20_ complex due to monomeric equivalence (1:1) between CPT and dC_20_ (for which the best templating ability is achieved as previously observed^9^). However, despite the evidence presented, both the fact that CPT has no IR/Raman bands in this region (Figure S11) and similarities with i-motif FTIR, we are unable to draw conclusions at this stage regarding the nature of the dC_20_ structure at pH 7 in the complex, but recognize that the TRIR data for the oligonucleotide alone support the formation of an i-motif type structure due to partial protonation at pH 7, which is in agreement with our previous CD measurements.^9^ In addition, two known transient bands for polycytosines are clearly observed, in the 1545 and 1585 cm^–1^ region, where the latter can be deconvoluted into 1574 and 1590 cm^–1^ bands. According to the literature, the characteristic transient band at 1574 cm^–1^ has only been observed previously in the spectra of nucleotides and polymeric strands but not in those of the single base, and has been attributed to the carbonyl stretching mode in the n_O_π* state of dC_20_.^45−47^ The band at 1545 cm^–1^ was previously assigned to a charge transfer between neighboring protonated and nonprotonated cytosines (C-:CH+) in the i-motif structure.^46^ One possibility is that this cytosine band in the TRIR spectra of the CPT/dC_20_ complex could be due to charge transfer between the thiophene and the cytosine (see below).

Returning to the focus of this work and the nature of CPT and oligonucleotide interactions, close observation of the TRIR spectra of CPT/dC_20_ with excitation at 266 nm in the region of 1550 to 1570 cm^–1^ shows some spectral evolution. Deconvolution of this region (Figure S12) revealed another transient at 1558 cm^–1^. According to the cytosine literature,^48^ this band most likely corresponds to the formation of the cytosine anion, which could be another marker for the formation of a charge transfer state between the polymer and the ssDNA. The redox potentials of thiophene (ionization potential (IP), 4.85 eV; electron affinity (EA), 3.15 eV)^49^ and cytosine (IP, 8.68 eV; EA, 0.56 eV)^50^ confirm the favored direction of oxidation, with an electron transferring from thiophene to cytosine, with the π-stacking between thiophene and cytosine facilitating the charge transfer.^48,51,52^ This new band at 1558 cm^–1^, which corresponds to a carbonyl stretching mode of the cytosine, decays with lifetime of ∼3 ps and exhibits a 5 cm^–1^ downshift within the first ∼10 ps (Figure S13a,b, respectively), possibly due to a vibrational Stark effect^53^ upon charge recombination, as this band is very sensitive to the electric environment. The shorter decay lifetime for this band, with respect to the shift dynamics, could be attributed to the noise in the extracted intensities from the deconvolution.

Two other intense negative bands stand out at 1498 and 1627 cm^–1^ in the TRIR spectrum of CPT/dC_20_. The band at 1498 cm^–1^ is attributed to a Fano antiresonance of the same C=C symmetric stretch of the thiophene ring in the polymer as seen with excitation at 532 nm (Figure 2a) facilitated by the overlap with the polaron electronic transition. The temporal behavior of this band is similar to the baseline decay, with a 3 cm^–1^ shift with time. The assignment is further supported by the appearance of this FA in the spectra of CPT alone with an excitation at 266 nm (Figure S8). The band located at 1627 cm^–1^, however, cannot be assigned to any expected bands from the polymer. The highest wavenumber vibrations predicted for CPT from DFT calculations (Figure S11, Table S2) are various imidazole ring vibrations, and these fall in the 1570 cm^–1^ region as seen from the ATR-FTIR spectrum of the polymer (Figure S10). These bands do not carry any strong intensity in the Raman spectrum to lead to noticeable Fano antiresonances (see Figure S14), in addition to the fact that they overlap strong transient bands in the TRIR spectra. Observing, however, the ATR-FTIR spectrum of CPT/dC_20_ (Figure S10), a band at ∼1618 cm^–1^ is visible as a shoulder to the more prominent water bend band at 1650 cm^–1^. Therefore, this band must correspond to a base vibration. Based on literature,^54,55^ the cytosine ring stretching vibration expected in this region is the ring mode stretch ν(C_5_=C_6_) + ν(N_3_=C_4_) *+ ν*(C_2_=O_7_). π–π stacking of the thiophene and cytosine rings in the complex found in our previous work^9^ followed by generation of the charge-transfer complex upon photoexcitation must perturb the environment of the base ring leading to enhanced bleach intensity of this band compared to the 266 nm excited TRIR spectra of dC_20_ alone. Interestingly this band is observed in the TRIR spectra of CPT/dC_20_ with excitation at 532 nm (Figure S15a) but not in the TRIR spectra of CPT alone at 532 or 266 nm (Figures S16 and S8b, respectively), further excluding this as a polymer vibration. This suggests that even without direct excitation of the ssDNA, because of π–π stacking between the base and thiophene rings and the generation of a charged species on the polymer chain, the ring environment is severely perturbed, leading to significant bleaching of the base ring vibration rather than the usually more strongly appearing carbonyl bands (Figure S15b). Similar signatures of DNA bleaching bands were observed in TRIR spectra of excited metal complexes bound to different DNA sequences and were used as indicators of the binding site on the DNA,^56,57^ or in cases of selective excitation of a cytosine base in a mixed oligonucleotide indicating the delocalization of charge between the different bases.^48^

The kinetics of bands (Table S3) associated with the base are very different in CPT/dC_20_ compared to those in dC_20_ alone, revealing the strong effect of complexation on ssDNA dynamics. Specifically, for dC_20_ alone, the 1574 cm^–1^ band exhibits slow kinetics on the scale of hundreds of ps and the 1545 cm^–1^ band decays even more slowly. Even though the kinetic traces of these two bands are noisy, the difference in the kinetics of these two bands (see Figures S17 and S18) is in line with the literature (lifetime of ∼150 ps for 1574 cm^–1^ and ∼300 ps for 1545 cm^–1^ band).^46^ Surprisingly, the respective cytosine bands when complexed with CPT recover much faster than expected (∼4 and 30 ps, respectively; Figures S19 and S20), suggesting that complexation contributes to faster relaxation of cytosine to the ground state. This explains the larger ground state population, evidenced by the transient peak at ∼1590 cm^–1^ due to “hot” ground state vibrational absorption and subsequent relaxation of dC_20_, which is more intense in the CPT/dC_20_ spectrum. The longer (∼30 ps) lifetime of the 1545 cm^–1^ band is also reflected in the dynamics of the 1627 cm^–1^ cytosine ring bleach band (Figure S15c), suggesting that the transient band could be associated with a ring vibration.

The occurrence of charge transfer between dC_20_ and the CPT is also evidenced by the existence of background absorption, attributed to a charge transfer state. The kinetics of the background and the vibrational bands were analyzed, revealing that the Fano antiresonance at 1498 cm^–1^ and the background absorption both undergo a double exponential decay with average time constants of 9 ± 2 ps for the former and 11 ± 1 ps for the latter respectively (Figures S21 and S22, Table S3), which suggests that they are associated with the same excited state process. These two markers combined with the formation of cytosine anion and its decay on a similar time scale (average time constant of 3.2 ± 1.8 ps, see Figure S13a), strongly support we are observing an electronic charge-transfer state in the mid-IR spectra. A low-energy charge transfer state was reported previously in the blends P3OT/C_60_ and MEH-PPV/C_60_ by Lee et al. at 0.2 eV (∼1600 cm^–1^), detected through PIA.^58^

In contrast to the case of the CPT/dC_20_ complex, TRIR measurements in CPT/dA_20_ and dA_20_ demonstrate a close similarity (Figure S23), as the spectra in both cases are consistent with the known peaks of adenosine reported in the literature, e.g., the intense bleach at ∼1630 cm^–1^ (C=N, C=C ring stretches),^59^ suggesting that the complex does not disturb the adenosine structure or dynamics. This is consistent with the weak binding between the polymer and dA_20_ as was implied by our previous UVRR results.^9^ In this case, we do not observe any evidence of charge transfer that could lead to new transient peaks due to the charged species, broad electronic absorption background, and Fano antiresonances of CPT such as the one at 1498 cm^–1^ in the TRIR spectra of CPT/dC_20_. Charge transfer must be restricted due to the absence of π-stacking interactions between thiophenes and adenines. In addition, the fact that the absorption of dA_20_ at 266 nm is five times larger than the absorption of CPT, explains the absence of any signal attributed to CPT excitation.

In summary, TRIR measurements on complexes of a CPT polymer with ssDNA provided greater detail on their photophysics and adds to our previous knowledge on the excited state behavior of such systems. We establish that both complexes, CPT/dC_20_ (a rigid complex with limited geometric relaxation) and CPT/dA_20_ (a flexible complex) form intrachain polarons following visible excitation. However, polaron formation is less for CPT/dA_20_ due to greater intrachain disorder and in this complex the polaron decays faster than the delocalized polaron formed in the case of CPT alone and the CPT/dC_20_ complex. The effective templating of CPT with ssDNA chains (such as dC_20_) through noncovalent interactions modifies the photophysical behavior of both partners. Figure 5 summarizes the differences; namely, UV excitation revealed modification of the excited state behavior of dC_20_, facilitated by the existence of numerous π-stacking interactions between the two components. A new band associated with the cytosine anion was observed, which combined with background absorption points to the formation of a charge transfer complex between CPT and dC_20_. This is in contrast to the CPT/dA_20_ case, where the TRIR spectra solely reflect the excited state behavior of the adenosine oligomer. In addition, a strong ground state bleach band associated with a cytosine ring band with or without excitation of the DNA reveals the perturbation of the cytosine ring environment due to the formation of charged species on the polymer backbone upon excitation and due to the π-stacking interactions between the cytosine and thiophene rings. Overall, these results suggest that while interactions between a scaffold and polymer are useful to control conformation, the scaffold, here being ssDNA, may not be a mere spectator but may influence excited state behavior. This key result highlights how one needs to consider potential effects that scaffolds have on the photophysics of templated conjugated polymers when designing such complexes for molecular electronics.

**Figure 5:**
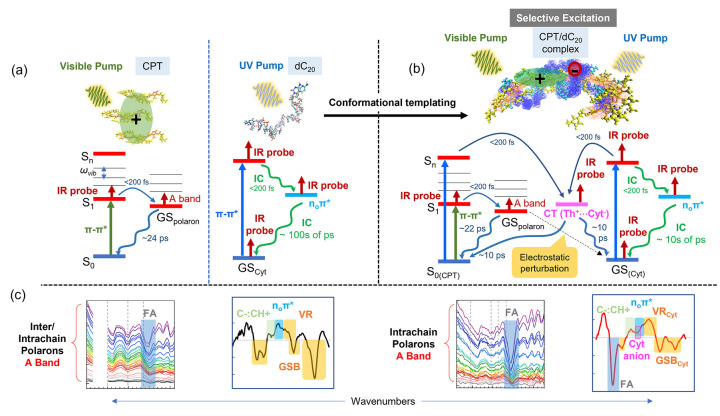
Simplified energy level diagram that describes the photophysics probed with TRIR in this study for (a) CPT and dC_20_ alone, and (b) the CPT/dC_20_ complex. Internal conversion (IC) from the excited state to the ground state of each system has been omitted for clarity. In part a, visible excitation of the CPT to the singlet excited state is followed by dissociation to free charges within the instrument response, with subsequent recombination back to the ground state. UV excitation of the π–π* transition of dC_20_ leads to IC to either the ground state (GS) followed by vibrational relaxation (VR) or to the n_O_π* state with subsequent IC to GS. In part b, selective excitation of either component of the complex reveals perturbation of the cytosine ring environment through electrostatic interactions. UV excitation of the complex leads to the generation of a CT state that severely modifies the dC_20_ dynamics. Red arrows indicate the various states probed with the IR pulse. (c) Indicative signatures observed in the TRIR spectra with either visible or UV excitation of the components alone (left) or the complex (right). The molecular conformations indicated were taken from MD simulations from our previous work.^9^

## Experimental Methods

*Materials and Sample Preparation.* Cationic poly(1*H*-imidazolium, 1-methyl-3-[2-[(4-methyl-3-thienyl)oxy]ethyl]-, chloride) (CPT), was synthesized by the Leclerc group (Université Laval). It has a molecular weight (*M*_w_) of 22 kDa (*M*_n_ = 11 kDa), with a polydispersity index (PDI) of 2.0, and the molecular weight of each monomer unit is 262.8 g/mol. A stock solution of the polymer was prepared in D_2_O and stored in the freezer. 1.2 mg of P3HT (*M*_w_ ∼ 50 kDa, PDI = 1.5) was dissolved in 1 mL of *d*-chloroform and then diluted to produce a solution with concentration of 1 mM. The single-stranded oligonucleotides (dA_20_ and dC_20_) were purchased from Sigma-Aldrich and a stock solution was prepared (in D_2_O) and stored in the freezer. Phosphate buffered saline (PBS) (pH 7.3, KH_2_PO_4_ 1.06 mM, Na_2_HPO_4_ 2.97 mM, NaCl 155 mM) was used to dilute the cationic polythiophene and ssDNA stock solutions to different monomeric concentrations depending on the ssDNA: 6 × 10^–4^ M for dA_20_ and for dC_20_. The order of addition for all of the solutions was the following: PBS (solvent), cationic polythiophene, and ssDNA.

*Time Resolved IR (TRIR) Spectroscopy.* Ps-TRIR measurements were performed at the LIFEtime setup, which is an ultrafast infrared absorption facility at the Rutherford Appleton Laboratory. Two 100 kHz Pharos lasers and three optical parametric amplifiers (Light Conversion Systems) provided one pump and two probe beams. The 266 and 532 nm excitations were performed with a narrow band, 220 fs-long pulse (150 cm^–1^ wide). The pump pulses at the sample have a fluence of 62 μJ/cm^2^ for 532 nm and 56.6 μJ/cm^2^ for 266 nm. The IR probe beams (pulse length of 180 fs (200 cm^–1^)) were dispersed in spectrographs and detected by MCT array detectors (IR Associates). The 50 kHz 532 nm pump pulses were focused (∼200 μm spot sizes) and overlapped with the probe beams (∼50 μm spot size) in the sample cell. The high-speed data acquisition system allowed 100 kHz acquisition and processing of the probe pulses to generate a pump-on-pump-off infrared absorption difference signal. The difference signal was calibrated using the characteristic polystyrene IR absorption spectrum..

Samples with an approximate volume of 0.8 mL were loaded onto a demountable liquid flow cell (Harrick Scientific Products, Inc.) comprised of two 25 mm-diameter CaF_2_ plates (Crystran Ltd.), separated by a 100-μm thick Teflon Spacer. In all experiments, the sample was raster-scanned in *x*- and *y*- directions and constantly recirculated using a peristaltic pump in order to preserve the integrity of the sample.

Details of processing of TRIR spectra and kinetic fitting can be found in the Supporting Information.

*ATR-FTIR Spectroscopy.* The FTIR measurements were performed on a Vertex 70 FTIR spectrometer (Bruker Optics, Ettlingen, Germany), equipped with a single-reflection ZnSe ATR accessory (Pike Technologies, Madison WI, USA) and a DTGS detector (Bruker Optics, Ettlingen, Germany). Spectra were collected with Opus 7.0 software (Bruker Optics, Ettlingen, Germany). Samples of 1 × 10^–3^ M for ssDNA and CPT/ssDNA and of 7 × 10^–3^ M for CPT were deposited on the crystal and allowed to dry for ∼1 h until solvent was evaporated prior to the measurement. A background spectrum was recorded with a clean crystal before the start of the measurements. The background and sample spectra were acquired with 64 scans at an instrument resolution of 4 cm^–1^ over the spectral range between 400 to 4000 cm^–1^. The contribution of the PBS buffer was also subtracted from each sample spectrum. MATLAB and ORIGIN software was used for spectral treatment and analysis.

## Supporting Information Available

Absorption, resonance Raman and ATR-FTIR spectra, additional TRIR spectra with excitation at 532 and 266 nm, fitting of the kinetics, calculated IR and Raman spectra and vibrational mode assignments for CPT (PDF)
