# Supplementary material for: Unraveling Excited State Dynamics of a Single-Stranded DNA-Assembled Conjugated Polyelectrolyte
Source: J Phys Chem Lett. 2023 Oct 26;14(44):9794–803. doi: 10.1021/acs.jpclett.3c01803 (PMC10641883; doi:10.1021/acs.jpclett.3c01803)
Supplement: Supplementary file 1 — jz3c01803_si_001.pdf [file jz3c01803_si_001.pdf]

Supporting Information

Unravelling Excited State Dynamics of a Single-Stranded DNA-Assembled Conjugated Polyelectrolyte

Eliana Nicolaidou,† Anthony W. Parker,‡ Igor V. Sazanovich,‡ Michael Towrie,‡ Sophia C. Hayes†,*

†*Dept. of Chemistry, University of Cyprus, P.O. Box 20537, 1678 Nicosia, Cyprus*
‡*Central Laser Facility, Research Complex at Harwell, Science and Technology Facilities Council, Rutherford Appleton Laboratory, Harwell Oxford, Didcot, Oxfordshire OX11 0QX, UK.*

Contents

1. TRIR spectra with excitation at 532 nm 2
2. Fitting of kinetics for 532 nm excited TRIR spectra 5
3. TRIR spectra with excitation at 266 nm 9
4. DFT computations of vibrational spectra 11
5. Cytosine anion spectral region 12
6. Identification of the 1620 cm^{-1} band 13
7. Fitting of kinetics for 266 nm excited TRIR spectra 15
8. TRIR spectra of CPT/dA_{20} with excitation at 266 nm 18

1. TRIR spectra with excitation at 532 nm

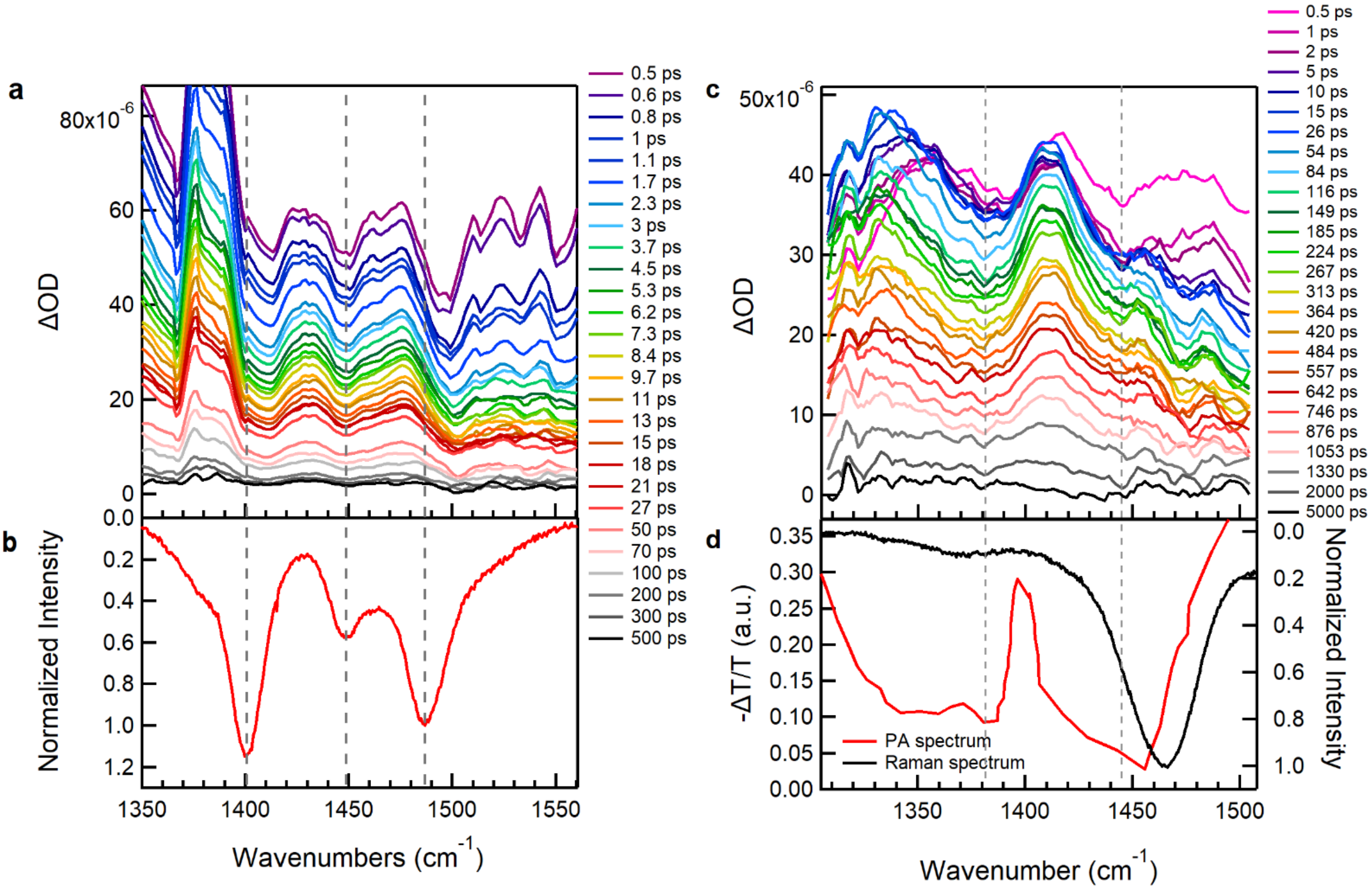

Figure S1. a) TRIR spectra of CPT in PBS buffer with excitation at 532 nm, without background subtraction. An artifact feature at ~ 1380 cm^{-1} is observed, which was generated by the detector in this data set and has been omitted from the data in the main text. b) Ground state Raman spectrum of CPT with excitation at 532 nm with inverted y axis. c) TRIR spectra of P3HT in d-chloroform (1 mM) without background subtraction with excitation at 532 nm. d) Photoinduced absorption (PA) in the mid-IR (red, left axis) reproduced from the literature (thin film)[1] and ground state resonance Raman spectrum of P3HT solution (black) with excitation at 532 nm with reversed y axis (black, right axis).

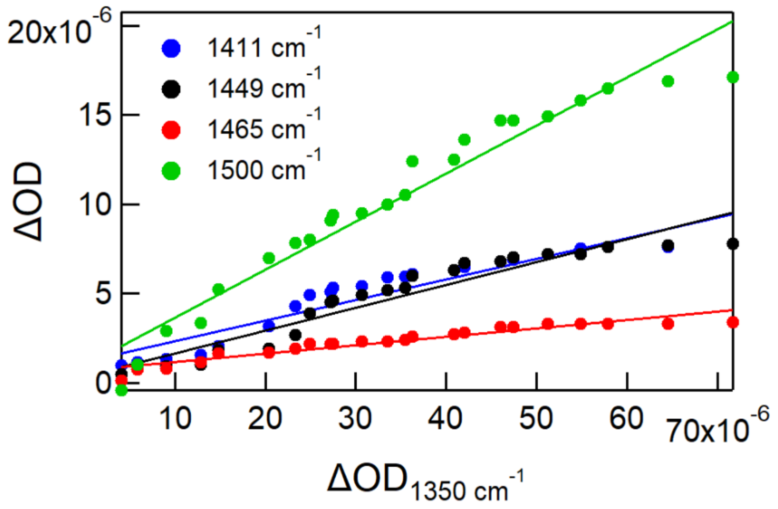

Figure S2. Linear correlation of the intensity of each vibrational band in the TRIR spectra of CPT alone and the intensity of a point (1350 cm^{-1}) considered as background as a function of delay time (between 1.7-200 ps).

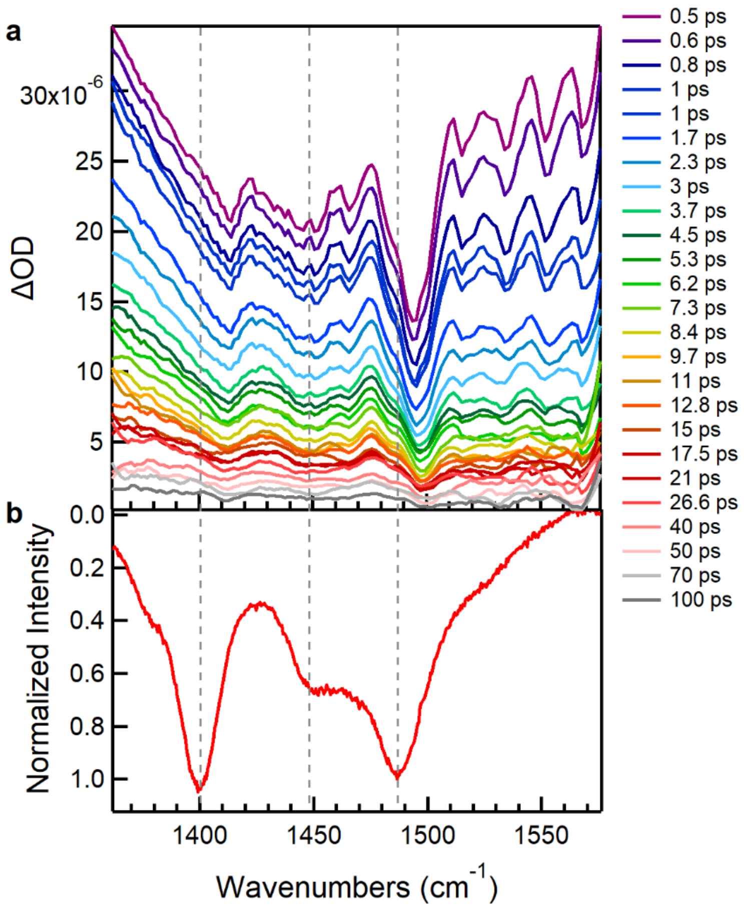

Figure S3. a) TRIR spectra of CPT/dA_{20} without background subtraction with excitation at 532 nm. b) Ground state Raman spectrum of CPT/dA_{20} with excitation at 473 nm with reversed y axis.

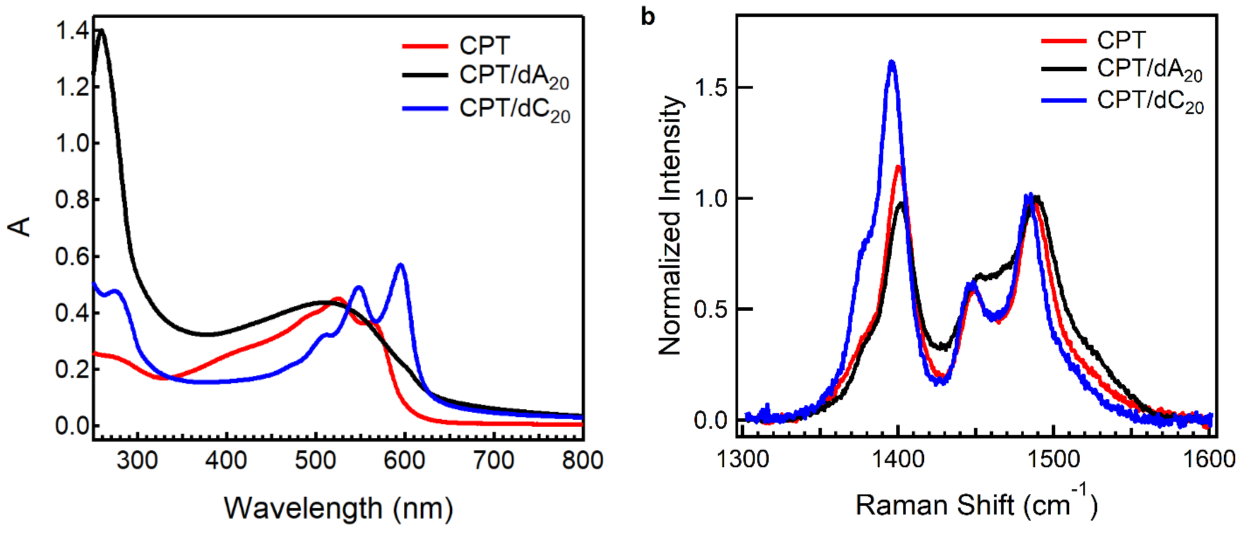

Figure S4. a) Absorption and b) Raman spectra (with excitation at 473 nm) of CPT (red) and its complexes with dA_{20} (black) and dC_{20} (blue).

2. Fitting of kinetics for 532 nm excited TRIR spectra

Single-point kinetics were extracted for the bands of interest using a point that corresponds to the maximum intensity of each band, which was then subtracted by the intensity of a point near the band considered as representative for the background (Figure S5-S6). The kinetics of the background were extracted by using the intensity of a point considered as representative for the background (Figure S7). The kinetics of the various features of the TRIR spectra were fit with a biexponential function (Equation 1) as seen in Figures S5-S7, S13, S15, S17 - S22.

$$f(t) = y_0 + A_1 exp\left(\frac{-(t-t_0)}{\tau_1}\right) + A_2 exp\left(\frac{-(t-t_0)}{\tau_2}\right) \quad (1)$$

Equation 2 was used for the calculation of the average time constant[2] (shown in Tables S1 and S3):

$$\tau_{avg} = \frac{\sum_i A_i \tau_i^2}{\sum_i A_i \tau_i} \quad (2)$$

where τ_i are the decay times extracted from double-exponential fits using Equation 1 and A_i the corresponding decay amplitudes.

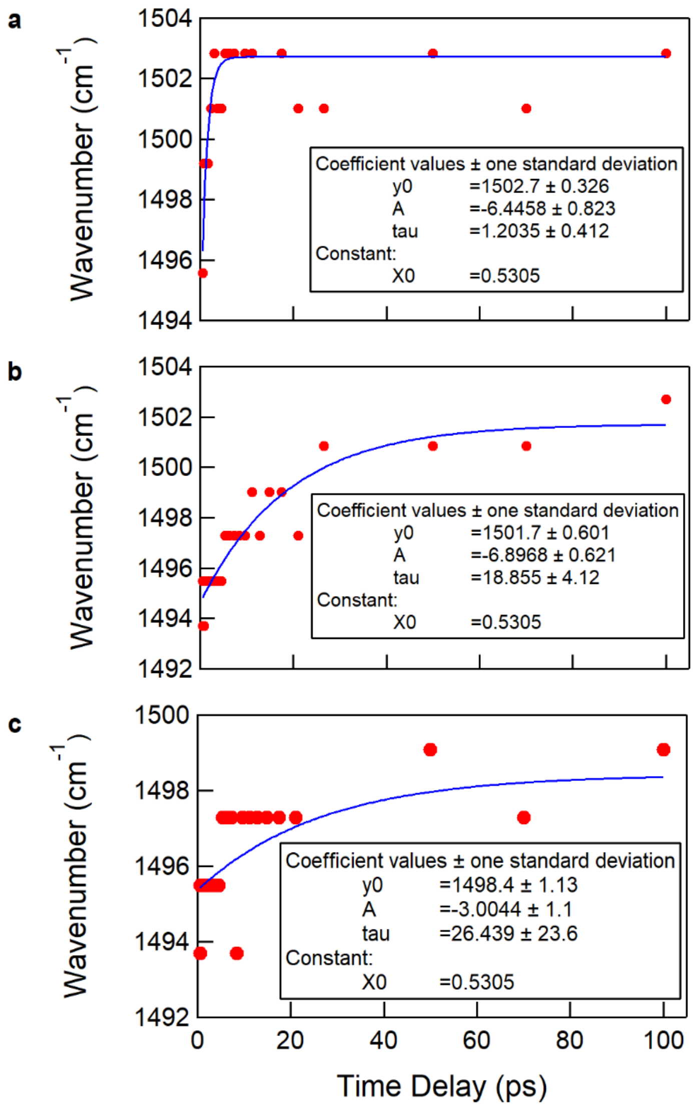

Figure S5. Kinetics of the shift of the 1498 cm^{-1} band of a) CPT alone, b) CPT/dA_{20} and c) CPT/dC_{20}. Background contribution was eliminated by subtracting the intensity of the ~1498 cm^{-1} band by the intensity of a point considered as representative for the background (1476 cm^{-1}).

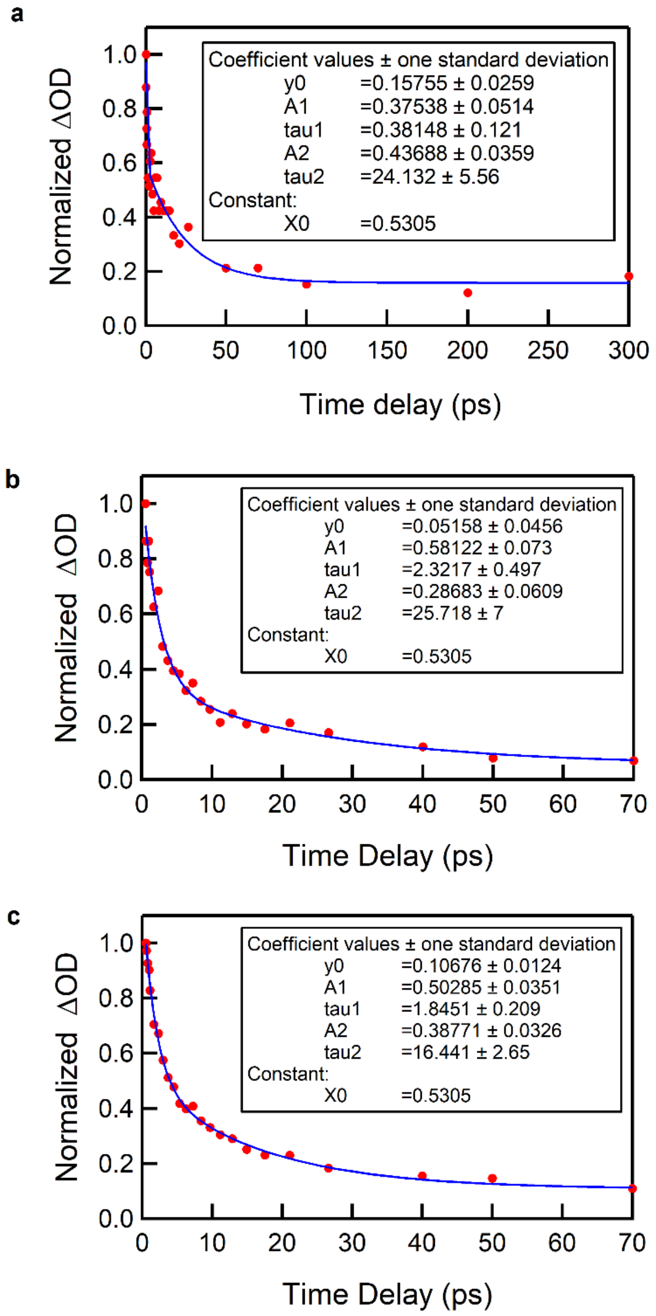

Figure S6. Kinetics of the 1498 cm^{-1} band of (a) CPT alone, (b) CPT/dC_{20}, and (c) CPT/dA_{20} after excitation at 532 nm fitted by a biexponential function. Background contribution was eliminated by subtracting the intensity of the ~1498 cm^{-1} band by the intensity of a point considered as representative for the background (1476 cm^{-1}).

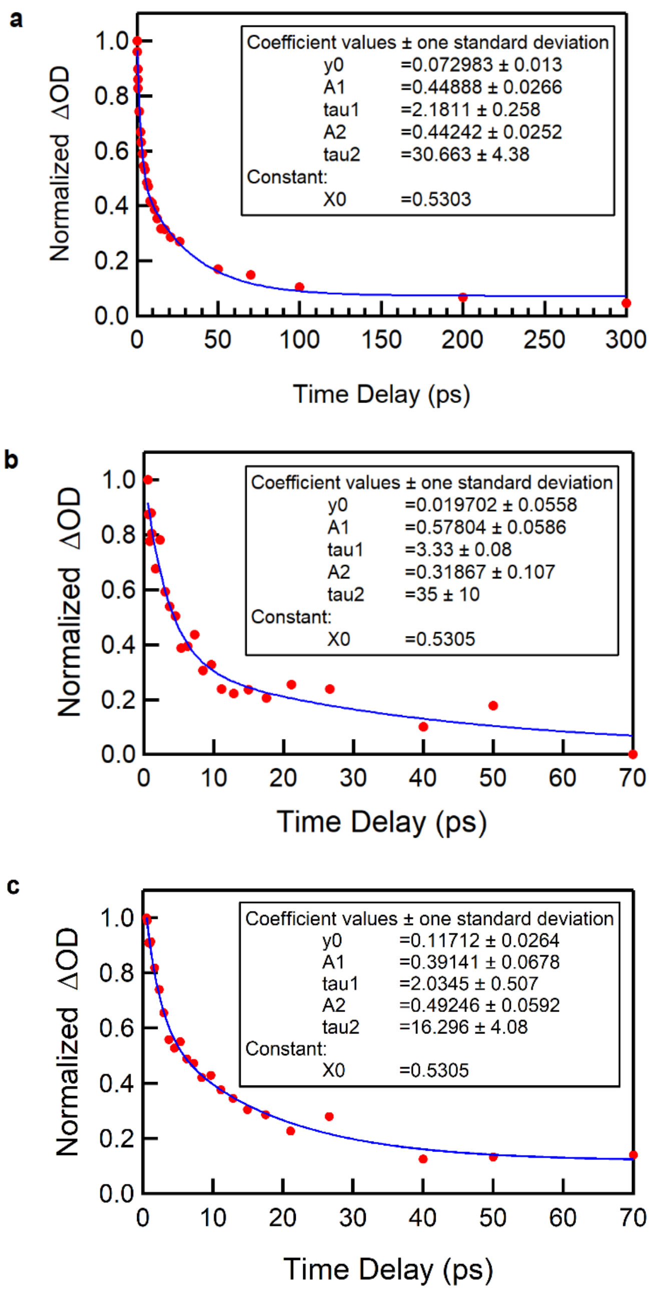

Figure S7. Kinetics of the background absorption of (a) CPT alone, (b) CPT/dC$_{20}$, and (c) CPT/dA$_{20}$ after excitation at 532 nm, fitted by a biexponential function. Representative points considered as background were at (a) 1350, (b) and (c) 1308 cm^{-1}.

Table S1. Time constants (τ_1, τ_2) obtained with a biexponential fit of the dynamics of background and the intensity of the 1498 cm^{-1} band for CPT alone, CPT/dC_{20} and CPT/dAC_{20} following 532 nm excitation.

	Background (ps)			1498 cm^{-1} band (ps)		
	τ_1	τ_2	τ_{avg}	τ_1	τ_2	τ_{avg}
**CPT**	2.2±0.2	31±4	29±4	0.4±0.1	24±6	24±6
**CPT/dC_{20}**	3.3±0.1	35±10	30±10	2.3±0.5	26±7	22±7
**CPT/dA_{20}**	2.0±0.5	16±4	15±4	1.8±0.2	16±3	15±3

3. TRIR spectra with excitation at 266 nm

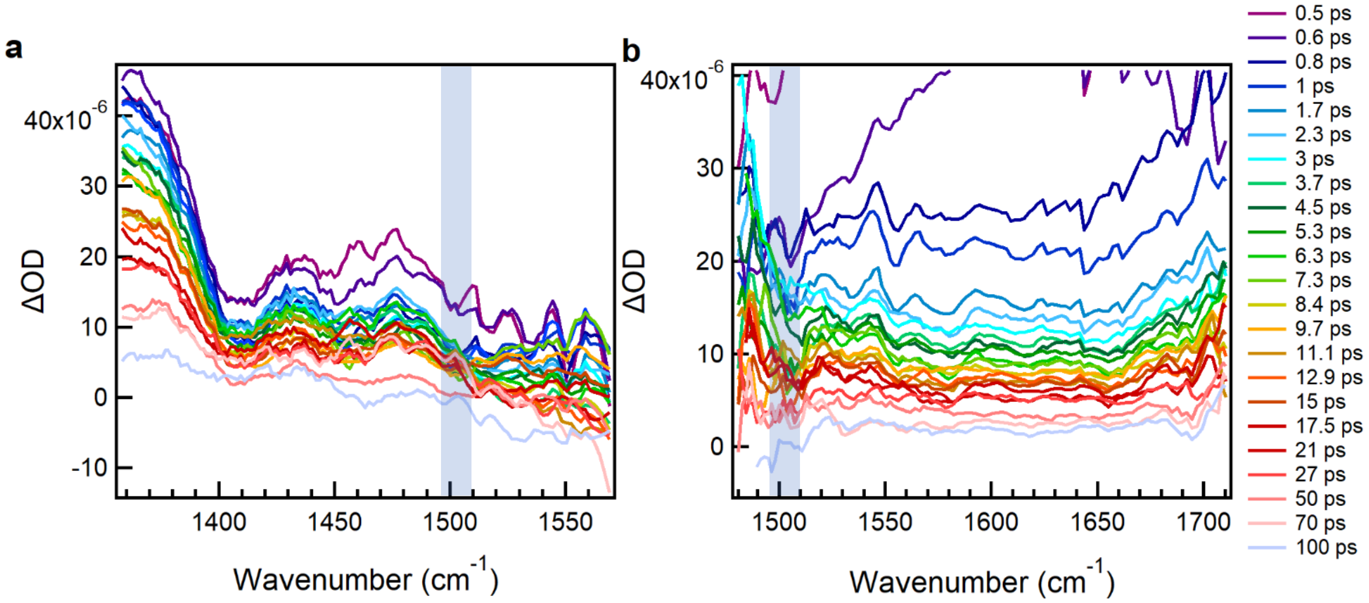

Figure S8. TRIR spectra of CPT alone without background subtraction with excitation at 266 nm at (a) lower and (b) higher wavenumbers. Shaded region indicates the Fano antiresonance (FA) of the thiophene C=C stretching mode. The lower FA intensity is attributed here to the lower background absorption compared to 532 nm and scales according to Figure S2.

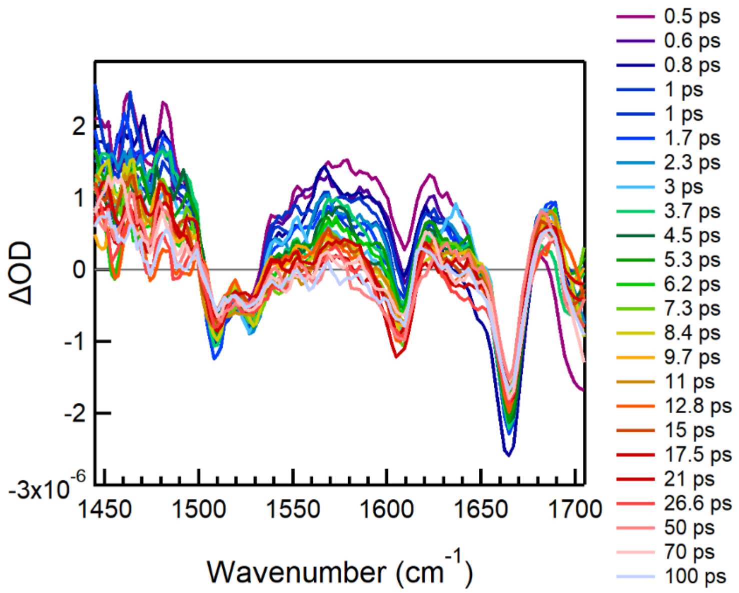

Figure S9. 266 nm excitation TRIR spectra of dC_{20} without background subtraction.

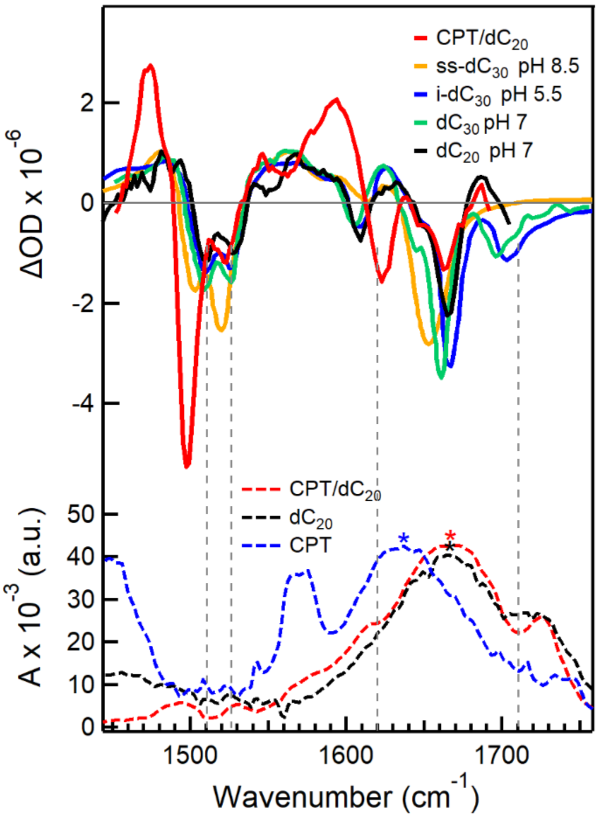

Figure S10. (Top) 266 nm excitation TRIR spectra after subtraction of background for CPT/dC_{20} (red) and dC_{20} at pH 7 (black) and dC_{30} at pH 8.5 (yellow), 5.5 (blue) and 7 (green) (TRIR spectra of dC_{30} were reproduced from literature[3]) at 2 ps. (Bottom) FTIR spectra of CPT/dC_{20} (red), dC_{20} (black) and CPT (blue). Water bending mode is marked with an asterix.

4. DFT computations of vibrational spectra

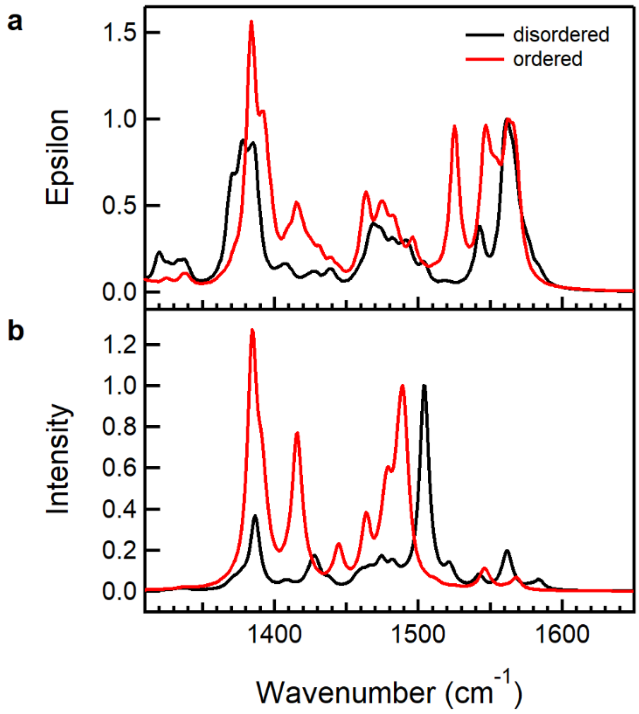

Figure S11. DFT calculated (a) IR (b) Raman spectra of disordered (black) and ordered (red) CPT chain (monomer units: n=4).[4]

Table S2. Assignment of experimental RR vibrational bands based on DFT calculated IR and Raman spectra. Computed frequencies refer to individual vibrational modes, and are corrected using the empirical scaling factor for B3LYP (0.97).[4]

Label	Frequency (cm^{-1})					Assignment
	Computed				Experimental	
	IR planar	IR twisted	Raman planar	Raman twisted	CPT/dC20 λ_{exc} = 532 nm	
ν_1	1384	1378	1384	1386	1376	Mixed C_β–$C_{\beta'}$ and C_α–$C_{\alpha'}$ stretching
ν_2	1415	1408	1416	1409	1394	Mixed C_β–$C_{\beta'}$ and C_α–$C_{\alpha'}$ stretching
ν_3	1464	1469	1445,1464	1428, 1466	1448, 1463	C_α=C_β(O) ring stretching
ν_4	1475, 1483	1483	1478, 1489	1474, 1504	1484	C_α=C_β(Me) ring stretching
ν_5	1525	-	1510	1522	1510	C_α=C_β ring stretching (anti)
ν_6	1547, 1562	1542, 1562	1546, 1567	1543, 1562, 1584	1625, 1650	C_α=C_β ring stretching (anti), C=C, C=N ring stretching (anti) (imidazole)

5. Cytosine anion spectral region

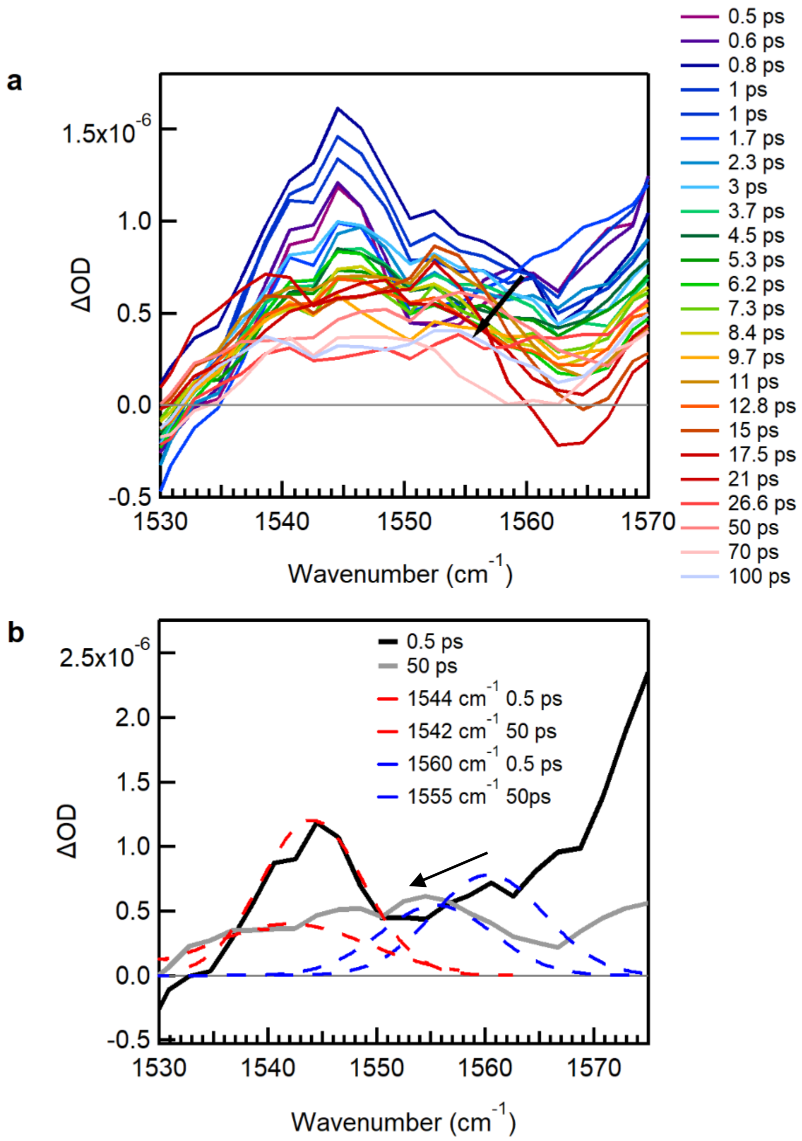

Figure S12. a) Spectral shift of 1558 cm^{-1} band with time indicated with a black arrow. b) Indicative deconvolution of the 1555 cm^{-1} spectral region for TRIR spectra at 0.5 ps (early) and 50 ps (later) delay times.

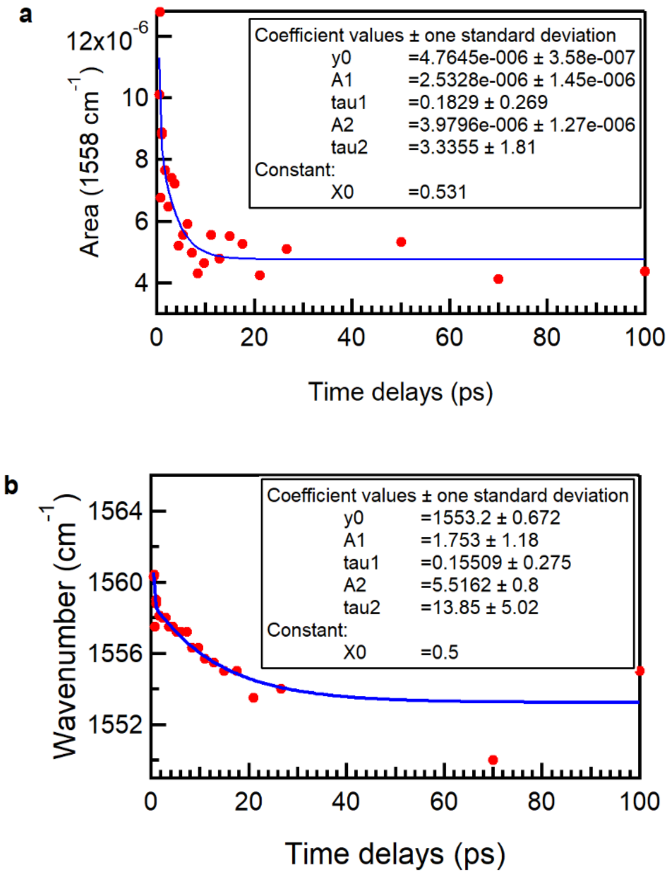

Figure S13. Kinetics of the 1558 cm^{-1} band a) area and b) shift in the TRIR spectra of CPT/dC_{20} after excitation at 266 nm, fitted by a biexponential function.

6. Identification of the 1620 cm^{-1} band

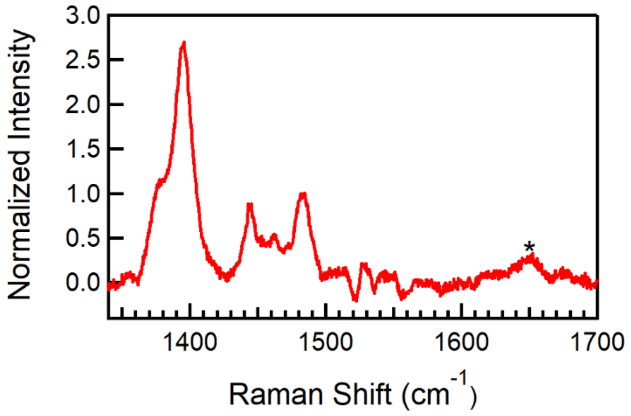

Figure S14. Resonance Raman spectrum of CPT/dC_{20} with excitation at 532 nm extended to higher wavenumbers. Water bend mode is marked with an asterisk.

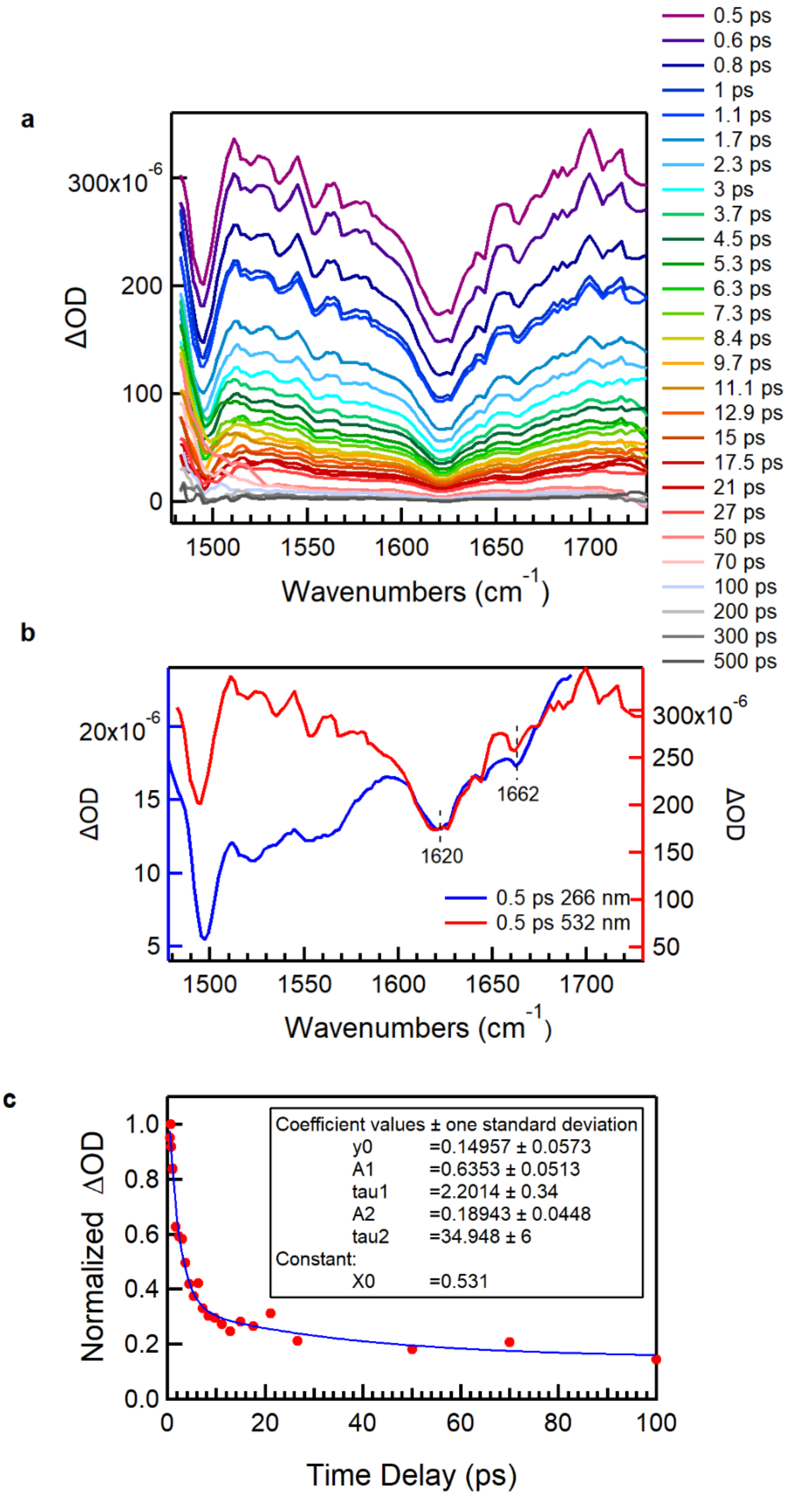

Figure S15. a) TRIR spectra of CPT/dC$_{20}$ without background subtraction with excitation at 532 nm at higher wavenumbers. b) Comparison of TRIR spectra of CPT/dC$_{20}$ at 0.5 ps with excitation at 266 nm (blue) and 532 nm (red). (c) Single-point kinetics of the 1622 cm^{-1} mode from the 266 nm excitation TRIR spectra of CPT/dC$_{20}$ after background subtraction of the spectra.

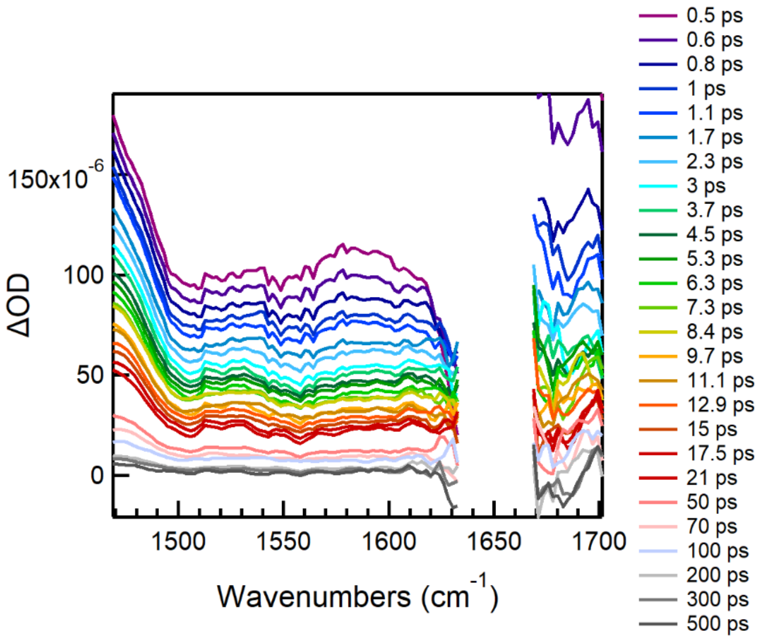

Figure S16. TRIR spectra of CPT alone without background subtraction with excitation at 532 nm at higher wavenumbers. There are no data between ~1633 and 1667 cm^{-1} due to complications with the detector (artifact). However, we believe that if the 1627 cm^{-1} band were present, this would be obvious already in the region starting ~1600 cm^{-1} as the FWHM was ~25 cm^{-1}.

7. Fitting of kinetics for 266 nm excited TRIR spectra

Single-point kinetics were extracted after background subtraction of the 266 nm excited TRIR spectra (using a polynomial fit to the baseline performed in MATLAB) and taking the maximum intensity of each band (Figures S17-S21). Single-point kinetics of the background were extracted from the polynomial fits to the TRIR spectra baseline. All the kinetics were then fitted using a biexponential function (Equation 1).

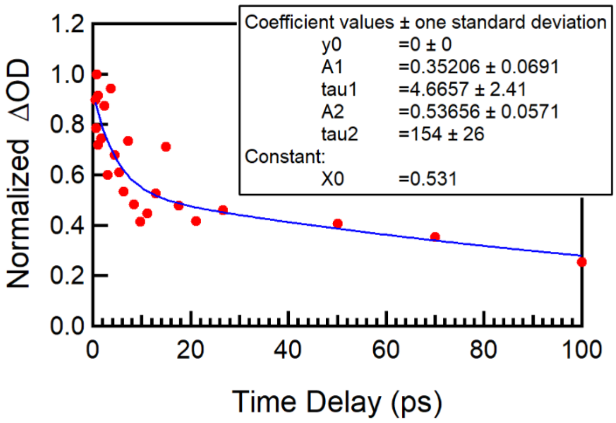

Figure S17. Kinetics of the 1569 cm^{-1} band of dC_{20} after excitation at 266 nm fitted by a biexponential function.

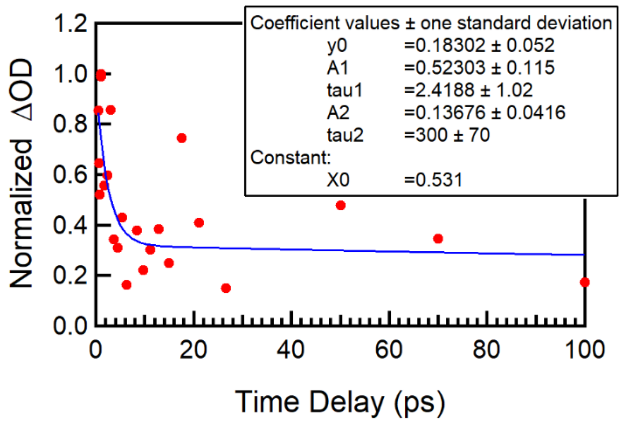

Figure S18. Kinetics of the 1542 cm^{-1} band of dC_{20} after excitation at 266 nm fitted by a biexponential function.

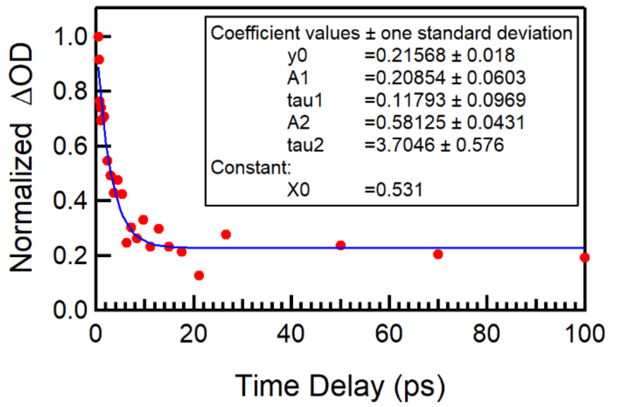

Figure S19. Kinetics of the 1577 cm^{-1} band of CPT/dC_{20} after excitation at 266 nm fitted by a biexponential function.

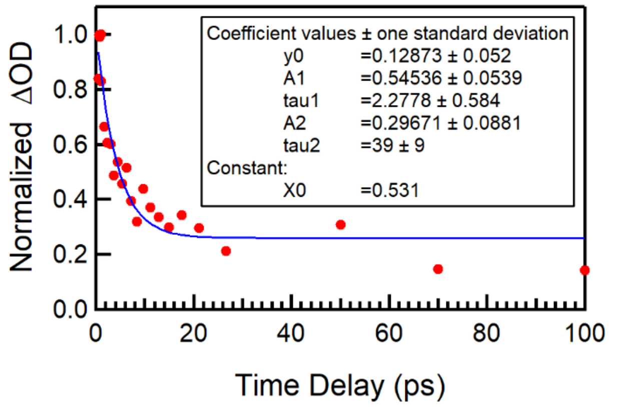

Figure S20. Kinetics of the 1544 cm^{-1} band of CPT/dC_{20} after excitation at 266 nm fitted by a biexponential function.

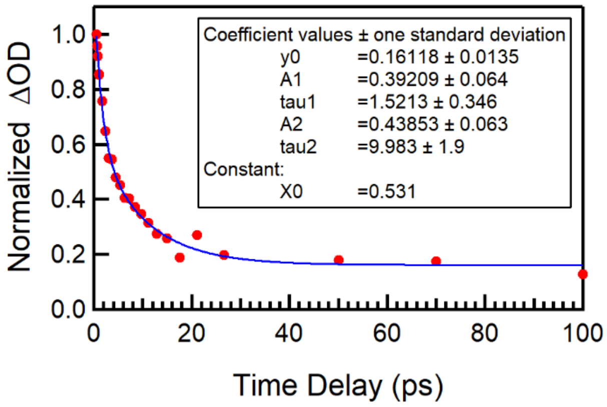

Figure S21. Kinetics of the 1498 cm^{-1} band of CPT/dC_{20} after excitation at 266 nm fitted by a biexponential function.

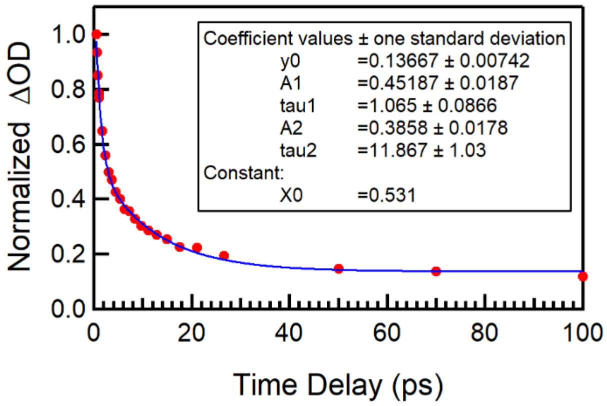

Figure S22. Kinetics of the background absorption of CPT/dC_{20} after excitation at 266 nm fitted by a biexponential function.

Table S3. Time constants (τ_1, τ_2) obtained with a biexponential global fit of dynamics of background and the intensity of bands for dC_{20} and CPT/dC_{20} following 266 nm excitation.

	dC_{20}			CPT/dC_{20}		
	τ_1	τ_2	τ_{avg}	τ_1	τ_2	τ_{avg}
Background (ps)	-	-	-	0.45±0.01	12±1	11±1
1498 cm^{-1} band (ps)	-	-	-	1.5±0.3	10±2	9±2
1545 cm^{-1} band (ps)	2.4±1.0	300±70	290±70	0.5±0.1	39±9	35±10
1558 cm^{-1} band (ps)	-	-	-	0.2±0.2	3.3±1.8	3.2±1.8
1577 cm^{-1} band (ps)	4.7±2.4	150±30	150±30	0.1±0.1	3.7±0.6	3.7±0.6
1627 cm^{-1} band (ps)	-	-	-	2.2±0.3	35±6	29±6

8. TRIR spectra of CPT/dA_{20} with excitation at 266 nm

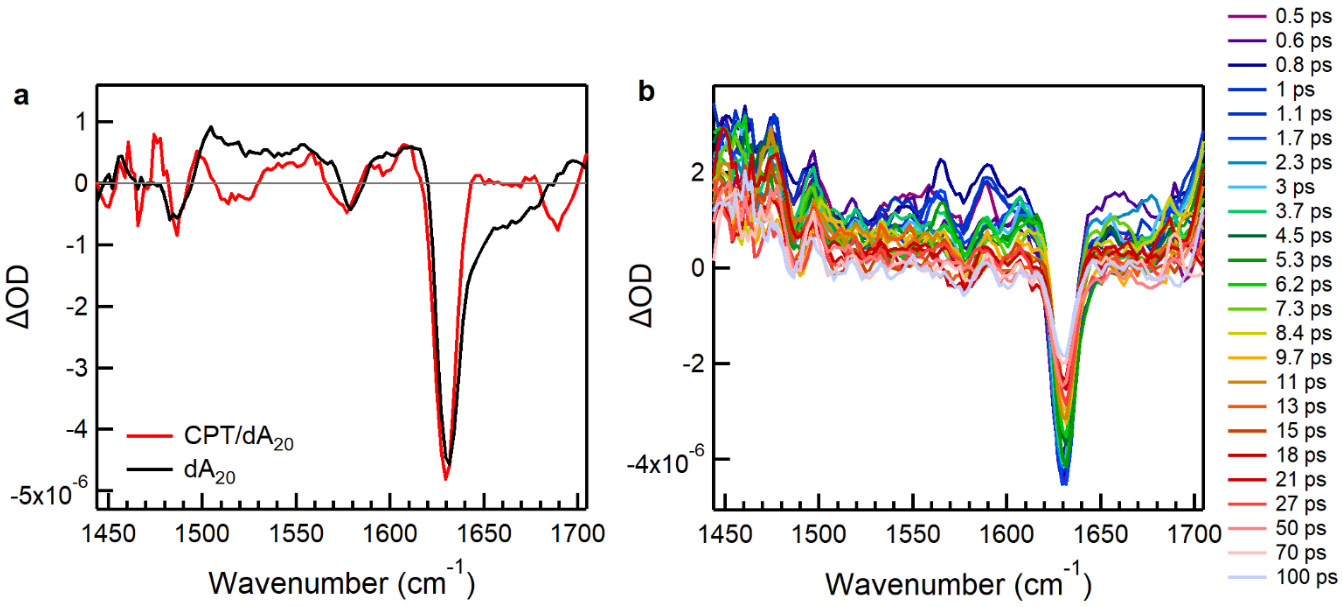

Figure S23. (a) TRIR spectra after the subtraction of the background of CPT/dA_{20} (red) and dA_{20} at pH 7 (black) at 2 ps (b) TRIR spectra of CPT/dA_{20} without background subtraction with excitation at 266 nm.

References

(1) Österbacka, R.; Jiang, X. M.; An, C. P.; Horovitz, B.; Vardeny, Z. V. Photoinduced Quantum Interference Antiresonances in π-Conjugated Polymers. *Phys. Rev. Lett.* **2002**, *88* (22), 4.

(2) Karecla, G.; Papagiorgis, P.; Panagi, N.; Zissimou, G. A.; Constantinides, C. P.; Koutentis, P. A.; Itskos, G.; Hayes, S. C. Emission from the Stable Blatter Radical. *New J. Chem.* **2017**, *41*, 8604–8613.

(3) Keane, P. M.; Wojdyla, M.; Doorley, G. W.; Kelly, J. M.; Parker, A. W.; Clark, I. P.; Greetham, G. M.; Towrie, M.; Magno, L. M.; Quinn, S. J. Long-Lived Excited States in i-Motif DNA Studied by Picosecond Time-Resolved IR Spectroscopy. *Chem. Commun.* **2014**, *50* (23), 2990–2992.

(4) Peterhans, L.; Alloa, E.; Sheima, Y.; Vannay, L.; Leclerc, M.; Corminboeuf, C.; Hayes, S. C.; Banerji, N. Salt-Induced Thermochromism of a Conjugated Polyelectrolyte. *Phys. Chem. Chem. Phys.* **2017**, *19* (42), 28853–28866.
